# Smartphone‐activated volunteer responders and survival to discharge after out‐of‐hospital cardiac arrests in Victoria, 2018–23: an observational cohort study

**DOI:** 10.5694/mja2.52673

**Published:** 2025-05-19

**Authors:** Belinda Delardes, Mads Christian Tofte Gregers, Emily Nehme, Michael Ray, Dylan Hall, Tony Walker, David Anderson, Daniel Okyere, Ashanti Dantanarayana, Ziad Nehme

**Affiliations:** ^1^ Centre for Research and Evaluation Ambulance Victoria Melbourne VIC; ^2^ Copenhagen University Hospital København Denmark; ^3^ Herlev and Gentofte University Hospital København Denmark; ^4^ Monash University Melbourne VIC; ^5^ Alfred Health Melbourne VIC; ^6^ Ambulance Victoria Melbourne VIC

**Keywords:** Resuscitation, First aid

## Abstract

**Objectives:**

To compare the likelihood of patient survival to discharge and of bystander cardiopulmonary resuscitation (CPR) and defibrillation for cases of out‐of‐hospital cardiac arrest in which at least one smartphone‐activated volunteer responder (SAVR) arrived before emergency medical services (EMS) with cases in which EMS arrived first.

**Study design:**

Population‐based observational cohort study; analysis of Victorian Ambulance Cardiac Arrest Registry (VACAR) data.

**Setting:**

Victoria, 12 February 2018 – 31 August 2023.

**Participants:**

All cases of out‐of‐hospital cardiac arrest not witnessed by EMS personnel, except events in residential aged care facilities, in which EMS personnel did not attempt resuscitation, or for which the EMS dispatch code was ineligible for SAVR activation; events during coronavirus disease 2019 pandemic lockdowns were also excluded (SAVR program pause: rural areas: 23 March 2020 – 16 October 2020; metropolitan areas: 23 March 2020 – 9 November 2020).

**Main outcome measures:**

Primary outcome: survival to hospital discharge. Secondary outcomes: bystander CPR, bystander defibrillation, any return of spontaneous circulation.

**Results:**

Of 9196 cases of out‐of‐hospital cardiac arrest included in our analysis, 1158 (12.6%) had been attended by SAVRs: before EMS arrival in 564 cases (48.7%) and after EMS arrival in 594 cases (51.3%). The risk‐adjusted odds of patient survival to hospital discharge were higher for events in which SAVRs arrived before EMS than for those not attended by SAVRs (adjusted odds ratio [aOR], 1.37; 95% confidence interval [CI], 1.02–1.85), as were those of bystander CPR (aOR, 7.59; 95% CI, 4.97–11.6) and bystander defibrillation (aOR, 16.0; 95% CI, 9.23–27.7); the likelihood of return of spontaneous circulation was similar for the two event groups. SAVRs arriving after EMS did not influence any of the assessed outcomes.

**Conclusion:**

The arrival of SAVRs before EMS personnel was associated with greater likelihood of patient survival to hospital discharge and of bystander CPR and defibrillation.

**The known**: Bystander cardiopulmonary resuscitation and defibrillation improve survival for people who experience out‐of‐hospital cardiac arrest.

**The new**: Survival to hospital discharge was 37% more likely (risk‐adjusted) after out‐of‐hospital cardiac arrest if smartphone‐activated volunteer responders arrived before emergency medical services, but it was not influenced if they arrived after the paramedics.

**The implications**: Smartphone‐activated volunteer responder programs could increase the likelihood of survival after out‐of‐hospital cardiac arrest if responders arrive before emergency medical services. Volunteer response times should be improved, and safeguards developed for limiting the exposure of volunteer responders to distressing cases, particularly for responders arriving after paramedics.

Out‐of‐hospital cardiac arrest is a leading cause of death worldwide.^1^, ^2^ Rapid intervention by bystanders who can provide cardiopulmonary resuscitation (CPR) or defibrillation using publicly available automated external defibrillators is associated with increased survival after out‐of‐hospital cardiac arrest.^3^, ^4^, ^5^ To increase bystander CPR and defibrillation rates, the American Heart Association,^6^ the Global Resuscitation Alliance,^7^ and the European Resuscitation Council recommend volunteer responder programs.^8^ In these programs, members of the community are alerted to nearby out‐of‐hospital cardiac arrests; in most programs, both off duty health care staff and laypeople can register as volunteer responders. Most current volunteer responder programs are based on smartphone technology that locates volunteers using the Global Positioning System (GPS).^9^

In Victoria, a smartphone‐activated volunteer responder (SAVR) program has operated since 2018.^10^ To compare the Victorian SAVR program with overseas systems, we compared the likelihood of bystander CPR, defibrillation, and survival of patients to discharge for cases of out‐of‐hospital cardiac arrest in which at least one SAVR arrived before emergency medical services (EMS) with cases in which EMS arrived first.

## Methods

We undertook a retrospective observational study of all out‐of‐hospital cardiac arrests not witnessed by EMS personnel that were eligible for SAVR activation in Victoria during 12 February 2018 – 31 August 2023. We excluded cardiac arrests in residential aged care facilities, and cases in which resuscitation by EMS personnel was not attempted. We also excluded cases during 23 March 2020 – 16 October 2020, when the Victorian SAVR program was paused because of coronavirus disease 2019 (COVID‐19) lockdowns; it recommenced in rural regions on 16 October 2020 and elsewhere on 9 November 2020. We also excluded cases with EMS dispatch codes ineligible for SAVR activation; that is, if the person taking the emergency call identified that the cardiac arrest is secondary to trauma, hanging, overdose, or assault, or if there are site‐related safety concerns, but the precipitating event is not always identified before EMS arrival. We reported our study according to the Strengthening the Reporting of Observational Studies in Epidemiology (STROBE) statement.^11^

### Emergency medical services in Victoria

Ambulance Victoria, the sole EMS operator in Victoria, uses the Medical Priority Dispatch System for ambulance call‐taking and dispatch.^12^ High priority cases, including suspected out‐of‐hospital cardiac arrests, trigger the dual dispatch of advanced life support and intensive care paramedics, and the emergency dispatcher provides CPR guidance to the caller by phone. In addition, SAVRs are alerted if eligible suspected out‐of‐hospital cardiac arrests are identified in the emergency call.

### The smartphone‐activated volunteer responder application

The Victorian SAVR program is managed using the GoodSAM app. The app, integrated with the emergency dispatch centre system, provides the GPS locations of potential SAVRs within a pre‐determined radius of the incident. To register as a GoodSAM responder, a person must be at least 18 years of age and provide a valid form of government identification. At the start of our study, the program accepted only registered health professionals and members of emergency service organisations. On 4 July 2019, the program expanded to accept any adult who had completed a nationally accredited first aid training certificate program; it was further expanded in December 2019 to accept any adult who reported the ability to provide CPR and use an automatic external defibrillator. The GoodSAM app includes information about registered publicly available automatic external defibrillators and displays them on a map for SAVRs to collect on their way to the incident. When activated, the GoodSAM app automatically locates and alerts the three nearest volunteer responders within 400 m (before 1 July 2021) or 500 m (since 1 July 2021) in metropolitan areas, and within 5 km of the event in regional areas. The GoodSAM app continues to alert SAVRs until three people from within the activation radius have accepted the alert. The emergency dispatch centre can cancel an alert if concerns about scene safety arise during the call or an SAVR is not required. SAVRs are not legally obliged to accept alerts.

### Data sources

We identified out‐of‐hospital cardiac arrests in the Victorian Ambulance Cardiac Arrest Registry (VACAR). Every out‐of‐hospital cardiac arrest attended by Ambulance Victoria is registered in the VACAR, using data from electronic patient care records;^13^ data are captured according to the Utstein Resuscitation Registry Template and entered into the VACAR by trained data processors.^14^ Information about whether SAVRs arrived before or after EMS was derived from follow‐up telephone interviews with responders who had accepted alerts, the GoodSAM application data transmitted to the Ambulance Victoria Clinical Data Warehouse, and electronic patient care records.

Additional information, including patient age (years), gender (male or female), remoteness (Greater Melbourne Metropolitan region or rural), presumed aetiology (medical or other, including trauma, asphyxiation, hanging), event location (private residence or other, including public place or workplace), witnessed status, year of arrest (categorical), and time from call to EMS arrival (continuous, in minutes) were obtained from the VACAR.

### Study exposure

We compared cases in which no SAVR arrived, those in which at least one SAVR arrived before EMS personnel, and those in which at least one SAVR arrived after EMS personnel.

### Study outcomes

The primary outcome was survival to hospital discharge. Secondary outcomes were bystander provision of CPR, bystander provision of defibrillation, and pre‐hospital return of spontaneous circulation. We could not determine whether the bystander who performed compressions and defibrillation in individual cases was the GoodSAM responder or another bystander.

### Statistical analysis

Categorical variables are summarised as numbers and proportions, continuous variables as medians with interquartile ranges (IQRs). The statistical significance of between‐group differences was determined in χ^2^ and Kruskal–Wallis tests; *P* < 0.05 was deemed statistically significant.

We assessed associations of SAVR arrival status with survival to hospital discharge, bystander CPR, bystander defibrillation (in initially shockable cases), and pre‐hospital return of spontaneous circulation in logistic regression analyses adjusted for patient age, gender, remoteness, presumed aetiology, event location, witnessed status, year of arrest, and time from call to EMS arrival. We report adjusted odds ratios (aORs) with 95% confidence intervals (CIs).

In a sensitivity analysis (propensity score‐matched cohort), we estimated propensity scores with a logistic regression model, with patient age, gender, remoteness, presumed aetiology, event location, witnessed status, and time from call to EMS arrival as predictor variables. Further, we stratified matching by year because survival after out‐of‐hospital cardiac arrests was influenced by the COVID‐19 pandemic. We used a nearest‐neighbour matching algorithm without replacement, with a 1:1 ratio and maximum calliper width of 0.01 to match people reached by an SAVR before EMS arrival with those who were not. After matching, covariate balance between treatment and control groups was assessed using standardised mean differences, with values less than 0.1 deemed to indicate adequate balance. The matched sample was then used to assess the association between SAVR status and bystander CPR, any return of spontaneous circulation, and survival to discharge in logistic regression analyses; we report odds ratios [ORs] with 95% CIs. Data limitations in the matched cohort prevented logistic regression analysis of the effect of SAVR status on bystander defibrillation; we therefore used the Fisher exact test to assess the statistical significance of differences in proportions in cases not attended by SAVRs and cases in which they arrived before EMS.

We used pairwise deletion to adjust for missing data. Statistical analyses were performed in Stata 17.0; propensity score matching was performed using the Stata *psmatch2* package.

### Ethics approval

The study was approved by the Monash University Human Research Ethics Committee (21046).

## Results

We included 9196 cases in our analysis, including 1158 (12.6%) attended by SAVRs (Box 1), before EMS personnel arrived in 564 cases (48.7%) and after EMS personnel arrived in 594 cases (51.3%).

Box 1 **Out‐of‐hospital cardiac arrests in Victoria, 12 February 2018 – 31 August 2023: selection of cases for inclusion in our analysis of the influence of smartphone‐activated volunteer responder (SAVR) attendance on patient outcomes**

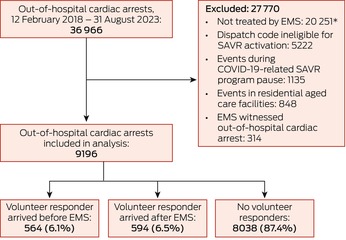

COVID‐19 = coronavirus disease 2019; EMS = emergency medical services personnel.

* No cardiopulmonary resuscitation or defibrillation, and no return of spontaneous circulation.

### Patient and event characteristics

The proportion of out‐of‐hospital cardiac arrests that occurred in metropolitan areas was larger for events not attended by SAVRs (5809, 72.3%) than for those in which SAVRs arrived before EMS (321, 56.9%). The median time from call to EMS arrival was longer for events in which SAVRs arrived before EMS (10.0 min; IQR, 7.8–13.7 min) than for events not attended by SAVRs (8.8 min; IQR, 7.0–11.4 min). The proportion of patients with initially shockable rhythms was larger for events in which SAVRs arrived before EMS (194, 34.6%) than in events not attended by SAVRs (2088, 26.0%). The proportion of events in which SAVRs arrived before EMS increased from 0.8% in 2018 to 12.8% in 2023 (Box 2).

The bystander CPR proportion was larger for events in which SAVRs arrived before EMS (540, 95.7%) than for events not attended by SAVRs (6018, 74.9%), as was the bystander defibrillation proportion (39, 20.1% *v* 35, 1.7%). The median time from the initial call to first defibrillation for patients with initially shockable rhythms was similar for all three groups, as were the proportions of people who experienced return of spontaneous circulation at any time or who survived to hospital discharge (Box 2).

Box 2 **Patient and event characteristics for out‐of‐hospital cardiac arrests in which emergency medical service (EMS) personnel attempted resuscitation, Victoria, 12 February 2018 – 31 August 2023**

**Table:** 

Characteristic	All events	No volunteer responders	Volunteer responder arrived after EMS	Volunteer responder arrived before EMS	Missing data	P*
Number of cases	9196	8038	594	564		
Age (years), median (IQR)	66 (52–78)	66 (51–77)	69 (55–78)	69 (55–78)	9	0.004
Gender (men)	6322 (68.8%)	5515 (68.6%)	394 (66.3%)	413 (73.2%)	1	0.031
Remoteness (metropolitan)	6470 (70.4%)	5809 (72.3%)	340 (57.2%)	321 (56.9%)	1	< 0.001
Event location (residence)	7380 (80.3%)	6483 (80.7%)	441 (74.2%)	456 (80.9%)	6	0.001
Witnessed	4588 (50.2%)	3945 (49.4%)	342 (57.6%)	301 (53.7%)	47	< 0.001
Presumed aetiology (medical)	8389 (91.2%)	7272 (90.5%)	569 (95.8%)	548 (97.2%)	0	< 0.001
Initial shockable rhythm	2456 (26.8%)	2088 (26.0%)	174 (29.4%)	194 (34.6%)	37	< 0.001
Time from call to EMS arrival (min), median (IQR)	8.9 (7.0–11.5)	8.8 (7.0–11.4)	8.6 (6.7–11.2)	10.0 (7.8–13.7)	6	< 0.001
Year^†^					0	< 0.001
2018	1652 (18.0%)	1582 [95.8%]	56 [3.4%]	14 [0.8%]		
2019	1909 (20.8%)	1705 [89.3%]	126 [6.6%]	78 [4.1%]		
2020	771 (8.4%)	675 [87.5%]	57 [7.4%]	39 [5.1%]		
2021	1955 (21.3%)	1703 [87.1%]	107 [5.5%]	145 [7.4%]		
2022	2079 (22.6%)	1746 [84.0%]	151 [7.3%]	182 [8.8%]		
2023	830 (9.0%)	627 [75.5%]	97 [11.7%]	106 [12.8%]		
Bystander cardiopulmonary resuscitation	7024 (76.4%)	6018 (74.9%)	466 (78.5%)	540 (95.7%)	0	< 0.001
First shock^‡^					268	< 0.001
Public access defibrillator	80 (3.7%)	35 (1.7%)	6 (3.9%)	39 (20.1%)		
Paramedics	1766 (80.7%)	1521 (82.0%)	128 (83.7%)	117 (65.0%)		
Fire service	342 (15.6%)	299 (16.1%)	19 (12.4%)	24 (13.3%)		
Call to first defibrillation^‡^ (min), median (IQR)	9.9 (7.9–12.4)	9.8 (7.9–12.3)	9.7 (7.6–12.7)	10.6 (8.3–13.6)	53	0.06
Any return of spontaneous circulation	3256 (35.4%)	2827 (35.2%)	220 (37.0%)	209 (37.1%)	0	0.46
Event survival	2722 (29.6%)	2366 (29.5%)	185 (31.1%)	171 (30.4%)	8	0.63
Survival to hospital discharge	1029 (11.3%)	880 (11.0%)	79 (13.5%)	70 (12.6%)	74	0.12

IQR = interquartile range.

* χ^2^ test for no volunteer responders *v* volunteer responder arrived after EMS *v* volunteer responder arrived before EMS.

† Row proportions provided for SAVR categories for each year.

‡ Patients with initially shockable rhythms only.

### Bystander cardiopulmonary resuscitation and defibrillation

Compared with events not attended by SAVRs, the risk‐adjusted odds of bystander CPR (aOR, 7.59; 95% CI, 4.97–11.6) and bystander defibrillation (aOR, 16.0; 95% CI, 9.23–27.7) were higher for events in which SAVRs arrived before EMS. For events in which the SAVR arrived after EMS, the odds of bystander CPR and defibrillation were not significantly different from those for events not attended by SAVRs (Box 3).

### Return of spontaneous circulation and survival to hospital discharge

The risk‐adjusted odds of pre‐hospital return of spontaneous circulation were similar for events attended by SAVRs (before or after EMS) and those that were not. The risk‐adjusted odds of survival to hospital discharge were higher for events in which SAVRs arrived before EMS than for those not attended by SAVRs (aOR, 1.37; 95% CI, 1.02–1.85) (Box 3). In the sensitivity analysis using the propensity score‐matched cohort, the odds of any return of spontaneous circulation (OR, 1.36; 95% CI, 1.06–1.74) and of survival to hospital discharge (OR, 1.56; 95% CI, 1.06–2.31) were each higher for events in which an SAVR arrived before EMS than for those not attended by SAVRs (Supporting Information, part 2).

Box 3 **Attendance by smartphone‐activated volunteer responders and bystander interventions and patient survival: multivariable logistic regression analyses**

**Table:** 

	Adjusted odds ratio (95% confidence interval)			
Characteristic	Bystander cardiopulmonary resuscitation	Bystander defibrillation*	Any return of spontaneous circulation	Survival to discharge
Smartphone‐activated volunteer responders^†^				
None	1	1	1	1
Arrived after emergency medical services	1.07 (0.87–1.32)	1.72 (0.70–4.22)	0.91 (0.75–1.10)	1.07 (0.81–1.41)
Arrived before emergency medical services	7.59 (4.97–11.6)	16.0 (9.23–27.7)	1.11 (0.91–1.35)	1.37 (1.02–1.85)
Age (per year)	0.99 (0.99–0.99)	1.02 (1.005–1.04)	0.99 (0.99–0.99)	0.97 (0.97–0.98)
Gender (men)	1.00 (0.90–1.11)	0.57 (0.31–1.03)	0.97 (0.88–1.07)	1.34 (1.13–1.59)
Remoteness (metropolitan)	0.70 (0.62–0.79)	1.11 (0.65–1.88)	1.07 (0.96–1.19)	0.95 (0.80–1.11)
Event location (residence)	0.52 (0.45–0.60)	0.23 (0.13–0.38)	0.48 (0.43–0.54)	0.24 (0.21–0.28)
Witnessed	1.77 (1.60–1.96)	2.37 (1.09–5.14)	4.82 (4.35–5.34)	6.86 (5.68–8.28)
Presumed aetiology (medical)	1.20 (0.99–1.44)	—	0.67 (0.56–0.80)	1.13 (0.84–1.50)
Time from call to EMS arrival (per minute)	1.00 (1.00–1.01)	1.04 (1.001–1.07)	0.95 (0.94–0.96)	0.92 (0.91–0.94)
Year				
2018	1	1	1	1
2019	1.10 (0.94–1.29)	1.21 (0.47–3.12)	1.11 (0.96–1.29)	1.01 (0.81–1.23)
2020	1.13 (0.91–1.39)	2.37 (0.83–6.75)	1.10 (0.91–1.34)	0.86 (0.64–1.16)
2021	0.90 (0.77–1.05)	0.92 (0.34–2.54)	0.97 (0.83–1.13)	0.77 (0.61–0.97)
2022	1.08 (0.92–1.26)	1.60 (0.64–3.97)	1.11 (0.96–1.29)	0.75 (0.60–0.93)
2023	0.99 (0.81–1.22)	1.42 (0.52–3.88)	1.31 (1.08–1.59)	0.72 (0.53–0.97)

* Patients with initially shockable rhythms only; precipitating event was medical in all such cases.

† The results of the unadjusted analysis are included in the Supporting Information, table 1.

## Discussion

In our observational study of 9196 cases of out‐of‐hospital cardiac arrests in Victoria eligible for SAVR activation via the GoodSAM application, we found that the risk‐adjusted odds of patient survival to hospital discharge, bystander CPR, and bystander defibrillation were higher for events in cases in which SAVRs arrived before EMS than for events not attended by SAVRs, but not for events in which SAVRs arrived after EMS personnel.

We found that the odds of survival to discharge, after adjustment for risk factors, were 37% higher for patients attended by a SAVR before EMS arrived. A recent meta‐analysis of one randomised controlled trial and eight observational studies similarly found that SAVR activation was associated with higher rates of survival at hospital discharge or 30 days (OR, 1.45; 95% CI, 1.21–1.74).^15^ However, the GRADE certainty of evidence level was moderate for the randomised controlled trial and very low for the observational studies. A second randomised controlled trial (in Sweden) could not proceed beyond phase one because of high group crossover, poor protocol compliance, and COVID‐19‐associated restrictions.^16^

We found that larger proportions of people who experienced out‐of‐hospital cardiac arrests received bystander CPR (95.7% *v* 74.9%) or had initially shockable rhythms (34.6% *v* 26.0%) in cases in which SAVRs arrived before EMS than in those not attended by SAVRs. Bystander CPR is associated with a greater likelihood of the patient presenting with an initially shockable rhythm^17^ and more robust initial ventricular fibrillation waveforms.^18^ As having an initially shockable rhythm is associated with increased likelihood of survival after out‐of‐hospital cardiac arrest,^19^ the benefit of SAVRs maintaining fibrillation with higher quality CPR may contribute to better survival rates.

### Smartphone‐activated volunteer responder programs

We found that SAVRs arriving before EMS was associated with a greater likelihood of bystander defibrillation, consistent with the findings of earlier observational studies.^20^, ^21^, ^22^, ^23^, ^24^ However, SAVR program dispatch systems are heterogenous, and consensus with respect to the optimal number of SAVRs to alert is limited.^10^ A recent Danish study found that several SAVRs arriving before the ambulance increased the likelihood of bystander defibrillation, and that it was most likely when three or more SAVRs arrived before the ambulance.^23^ The Danish SAVR program instead alerts a fixed number of SAVRs, regardless of whether they accept or decline the alert. In the Victorian SAVR program, the alert process continues until three SAVRs have accepted the alert, provided three are available within the defined activation radius. Alerting a fixed number of SAVRs is probably quicker, as second and later alerts are not contingent on SAVRs interacting with the alert, but continued alerting ensures that three SAVRs accept the alert if they are available.

The availability of both SAVRs and public defibrillators is generally lower in rural than metropolitan areas, which could attenuate the effect of SAVR programs.^25^, ^26^ Community‐based interventions, in which designated members of the community are instructed in CPR and defibrillator use, could increase SAVR recruitment and activity in these communities.^27^, ^28^ The combination in Singapore of community education with an SAVR program doubled the likelihood of survival after out‐of‐hospital cardiac arrest, but the generalisability of this effect is unclear.^29^

### Responder considerations

The GoodSAM app informs responders that their assistance is no longer required if EMS personnel have already arrived, but almost half the attending SAVRs in the cases included in our study arrived after EMS. We found no association between SAVRs arriving after EMS and patient survival. A Danish study reported that EMS staff found SAVRs helpful for providing continued assistance after EMS arrival,^30^ but collaboration between pre‐hospital clinicians and SAVRs could be difficult,^31^ and the presence of SAVRs after EMS arrival might be beneficial only when EMS resources are limited. The impact of SAVRs on the stress levels of pre‐hospital clinicians during out‐of‐hospital cardiac arrest resuscitations has not been investigated, but patients’ relatives have reported that their presence was comforting.^32^ SAVRs not trained in health care reported, on the other hand, that they felt underprepared to provide psychological support to relatives.^32^ We identified 41 instances of SAVRs responding to out‐of‐hospital cardiac arrests with non‐medical causes, including hangings, overdoses, and trauma, despite these categories being ineligible for SAVR dispatch. Unfortunately, distressed bystanders and family members calling EMS may be unable to provide the level of detail required to cancel SAVR activation, or this information can be received after SAVRs have arrived at the scene. Several studies have found that the risk of psychological harm for SAVRs is very low,^33^, ^34^, ^35^ but the risk should be mitigated if their presence does not improve patient outcomes, and follow‐up debriefing and psychological support should be offered.

### Limitations

Our retrospective observational study cannot establish causation. Several possible confounding factors, such as other medical conditions, variations in practitioner expertise, regional protocol differences, and access to tertiary care facilities could not be considered because this information was not available in the dataset, potentially influencing our effect estimates. Further, our primary outcome was survival to hospital discharge; neurological outcome could be a better marker of patient outcomes. The dataset did not include some potentially important details about SAVRs, including relevant medical training and expertise, or their exact time of arrival. As a result, we could not compare the effects of lay responders and off‐duty health care professionals as SAVRs, nor determine whether the effect of SAVR arrival on survival was arrival time‐sensitive. Finally, the generalisability of our findings outside Victoria is limited by the heterogeneity of SAVR programs.

### Conclusion

In our analysis of 9196 out‐of‐hospital cardiac arrests in Victoria during 2018–23, we found that the arrival of SAVRs before EMS was associated with greater likelihood of survival to hospital discharge, and of bystander CPR and defibrillation. SAVR arrival after EMS arrival did not influence these outcomes, and safeguards are needed to reduce the exposure of SAVRs to distressing situations in such cases. SAVR programs could increase the likelihood of survival for people who experience out‐of‐hospital cardiac arrest, and this possibility should be investigated in clinical trials.

## Open access

Open access publishing facilitated by Monash University, as part of the Wiley – Monash University agreement via the Council of Australian University Librarians.

## Competing interests

No relevant disclosures.

## Data sharing

The data that support the findings of this study are available from the corresponding author upon reasonable request. The data are not publicly available because of ethical and privacy restrictions.

Received 13 March 2024, accepted 25 November 2024.

## Supporting information


Supplementary results

## References

[1] Gräsner JT , Wnent J , Herlitz J , et al. Survival after out‐of‐hospital cardiac arrest in Europe: results of the EuReCa TWO study. Resuscitation 2020; 148: 218‐226. 32027980 10.1016/j.resuscitation.2019.12.042

[2] Virani SS , Alonso A , Benjamin EJ , et al; American Heart Association Council on Epidemiology and Prevention Statistics Committee and Stroke Statistics Subcommittee. Heart disease and stroke statistics: 2020 update. A report from the American Heart Association. Circulation 2020; 141: e139‐e596. 31992061 10.1161/CIR.0000000000000757

[3] Holmberg M , Holmberg S , Herlitz J ; Swedish Cardiac Arrest Registry. Factors modifying the effect of bystander cardiopulmonary resuscitation on survival in out‐of‐hospital cardiac arrest patients in Sweden. Eur Heart J 2001; 22: 511‐519. 11320981 10.1053/euhj.2000.2421

[4] Brooks SC , Clegg GR , Bray J , et al; International Liaison Committee on Resuscitation. Optimizing outcomes after out‐of‐hospital cardiac arrest with innovative approaches to public‐access defibrillation: a scientific statement from the International Liaison Committee on Resuscitation. Circulation 2022; 145: e776‐e801. 35164535 10.1161/CIR.0000000000001013

[5] Rajan S , Wissenberg M , Folke F , et al. Association of bystander cardiopulmonary resuscitation and survival according to ambulance response times after out‐of‐hospital cardiac arrest. Circulation 2016; 134: 2095‐2104. 27881566 10.1161/CIRCULATIONAHA.116.024400

[6] Berg KM , Cheng A , Panchal AR , et al; Adult Basic and Advanced Life Support, Pediatric Basic and Advanced Life Support, Neonatal Life Support, and Resuscitation Education Science Writing Groups. Part 7: Systems of care: 2020 American Heart Association guidelines for cardiopulmonary resuscitation and emergency cardiovascular care. Circulation 2020; 142 (16 Suppl 2): S580‐S604. 33081524 10.1161/CIR.0000000000000899

[7] Eisenberg M , Lippert F , Castren M , et al. Improving survival from out‐of‐hospital cardiac arrest. Acting on the call: 2018 update from the Global Resuscitation Alliance. Apr 2018. https://www.globalresuscitationalliance.org/wp‐content/pdf/acting_on_the_call.pdf (viewed Sept 2024).

[8] Semeraro F , Greif R , Böttiger BW , et al. European Resuscitation Council guidelines 2021: systems saving lives. Resuscitation 2021; 161: 80‐97. 33773834 10.1016/j.resuscitation.2021.02.008

[9] Valeriano A , Van Heer S , de Champlain F , Brooks SC . Crowdsourcing to save lives: a scoping review of bystander alert technologies for out‐of‐hospital cardiac arrest. Resuscitation 2021; 158: 94‐121. 33188832 10.1016/j.resuscitation.2020.10.035

[10] Smith CM , Wilson MH , Ghorbangholi A , et al. The use of trained volunteers in the response to out‐of‐hospital cardiac arrest: the GoodSAM experience. Resuscitation 2017; 121: 123‐126. 29079507 10.1016/j.resuscitation.2017.10.020

[11] Von Elm E , Altman DG , Egger M , et al; STROBE Initiative. The strengthening the reporting of observational studies in epidemiology (STROBE) statement: guidelines for reporting observational studies. Lancet 2007; 370: 1453‐1457. 18064739 10.1016/S0140-6736(07)61602-X

[12] Beck B , Bray JE , Smith K , et al; Aus‐ROC Steering Committee. Description of the ambulance services participating in the Aus‐ROC Australian and New Zealand out‐of‐hospital cardiac arrest epistry. Emerg Med Australas 2016; 28: 673‐683. 27728958 10.1111/1742-6723.12690

[13] Nehme Z , Bernard S , Cameron P , et al. Using a cardiac arrest registry to measure the quality of emergency medical service care: decade of findings from the Victorian Ambulance Cardiac Arrest Registry. Circ Cardiovasc Qual Outcomes 2015; 8: 56‐66. 25604556 10.1161/CIRCOUTCOMES.114.001185

[14] Perkins GD , Jacobs IG , Nadkarni VM , et al; Utstein Collaborators. Cardiac arrest and cardiopulmonary resuscitation outcome reports: update of the Utstein resuscitation registry templates for out‐of‐hospital cardiac arrest. Circulation 2015; 132: 1286‐1300. 25391522 10.1161/CIR.0000000000000144

[15] Scquizzato T , Belloni O , Semeraro F , et al. Dispatching citizens as first responders to out‐of‐hospital cardiac arrests: a systematic review and meta‐analysis. Eur J Emerg Med 2022; 29: 163‐172. 35283448 10.1097/MEJ.0000000000000915

[16] Berglund E , Hollenberg J , Jonsson M , et al. Effect of smartphone dispatch of volunteer responders on automated external defibrillators and out‐of‐hospital cardiac arrests: the SAMBA randomized clinical trial. JAMA Cardiol 2023; 8: 81‐88. 36449309 10.1001/jamacardio.2022.4362 PMC9713680

[17] Shibahashi K , Kato T , Hikone M , Sugiyama K . Bystander cardiopulmonary resuscitation and cardiac rhythm change over time in patients with out‐of‐hospital cardiac arrest. Emerg Med J 2023; 40: 418‐423. 37019616 10.1136/emermed-2022-212757

[18] Bessen B , Coult J , Blackwood J , et al. Insights from the ventricular fibrillation waveform into the mechanism of survival benefit from bystander cardiopulmonary resuscitation. J Am Heart Assoc 2021; 10: e020825. 34569292 10.1161/JAHA.121.020825 PMC8649127

[19] Amacher SA , Bohren C , Blatter R , et al. Long‐term survival after out‐of‐hospital cardiac arrest: a systematic review and meta‐analysis. JAMA Cardiol 2022; 7: 633‐643. 35507352 10.1001/jamacardio.2022.0795 PMC9069345

[20] Smith CM , Lall R , Fothergill RT , et al. The effect of the GoodSAM volunteer first‐responder app on survival to hospital discharge following out‐of‐hospital cardiac arrest. Eur Heart J Acute Cardiovasc Care 2022; 11: 20‐31. 35024801 10.1093/ehjacc/zuab103 PMC8757292

[21] Nielsen CG , Folke F , Andelius L , et al. Increased bystander intervention when volunteer responders attend out‐of‐hospital cardiac arrest. Front Cardiovasc Med 2022; 9: 1030843. 36407446 10.3389/fcvm.2022.1030843 PMC9672473

[22] Andelius L , Malta Hansen C , Lippert FK , et al. Smartphone activation of citizen responders to facilitate defibrillation in out‐of‐hospital cardiac arrest. J Am Coll Cardiol 2020; 76: 43‐53. 32616162 10.1016/j.jacc.2020.04.073

[23] Gregers MCT , Andelius L , Kjoelbye JS , et al. Association between number of volunteer responders and interventions before ambulance arrival for cardiac arrest. J Am Coll Cardiol 2023; 81: 668‐680. 36792282 10.1016/j.jacc.2022.11.047

[24] Stieglis R , Zijlstra JA , Riedijk F , et al. Alert system‐supported lay defibrillation and basic life‐support for cardiac arrest at home. Eur Heart J 2022; 43: 1465‐1474. 34791171 10.1093/eurheartj/ehab802 PMC9009403

[25] Stieglis R , Zijlstra JA , Riedijk F , et al. AED and text message responders density in residential areas for rapid response in out‐of‐hospital cardiac arrest. Resuscitation 2020; 150: 170‐177. 32045663 10.1016/j.resuscitation.2020.01.031

[26] Dicker B , Garrett N , Wong S , et al. Relationship between socioeconomic factors, distribution of public access defibrillators and incidence of out‐of‐hospital cardiac arrest. Resuscitation 2019; 138: 53‐58. 30802556 10.1016/j.resuscitation.2019.02.022

[27] Nielsen AM , Isbye DL , Lippert FK , Rasmussen LS . Can mass education and a television campaign change the attitudes towards cardiopulmonary resuscitation in a rural community? Scand J Trauma Resusc Emerg Med 2013; 21: 39. 23675991 10.1186/1757-7241-21-39 PMC3666962

[28] Boland LL , Formanek MB , Harkins KK , et al. Minnesota Heart Safe communities: are community‐based initiatives increasing pre‐ambulance CPR and AED use? Resuscitation 2017; 119: 33‐36. 28774567 10.1016/j.resuscitation.2017.07.031

[29] Tay PJM , Pek PP , Fan Q , et al. Effectiveness of a community based out‐of‐hospital cardiac arrest (OHCA) interventional bundle: results of a pilot study. Resuscitation 2020; 146: 220‐228. 31669756 10.1016/j.resuscitation.2019.10.015

[30] Jellestad ASL , Folke F , Molin R , et al. Collaboration between emergency physicians and citizen responders in out‐of‐hospital cardiac arrest resuscitation. Scand J Trauma Resusc Emerg Med 2021; 29: 110. 34344415 10.1186/s13049-021-00927-w PMC8330065

[31] Sørensen OB , Milling L , Laerkner E , et al. Professional prehospital clinicians’ experiences of ethical challenges associated with the collaboration with organised voluntary first responders: a qualitative study. Scand J Trauma Resusc Emerg Med 2023; 31: 79. 37964364 10.1186/s13049-023-01147-0 PMC10644536

[32] Kragh AR , Grabmayr AJ , Tjørnhøj‐Thomsen T , et al. Volunteer responder provision of support to relatives of out‐of‐hospital cardiac arrest patients: a qualitative study. BMJ Open 2023; 13: e071220. 10.1136/bmjopen-2022-071220 PMC10032384 36944472

[33] Kragh AR , Andelius L , Gregers MT , et al. Immediate psychological impact on citizen responders dispatched through a mobile application to out‐of‐hospital cardiac arrests. Resusc Plus 2021; 7: 100155. 34430949 10.1016/j.resplu.2021.100155 PMC8371246

[34] Ries ES , Kragh AR , Dammeyer J , et al. Association of psychological distress, contextual factors, and individual differences among citizen responders. J Am Heart Assoc 2021; 10: e020378. 34212765 10.1161/JAHA.120.020378 PMC8403282

[35] Haskins B , Nehme Z , Dicker B , et al. A binational survey of smartphone activated volunteer responders for out‐of‐hospital cardiac arrest: availability, interventions, and post‐traumatic stress. Resuscitation 2021; 169: 67‐75. 34710547 10.1016/j.resuscitation.2021.10.030

