# Supplementary material for: Smartphone‐activated volunteer responders and survival to discharge after out‐of‐hospital cardiac arrests in Victoria, 2018–23: an observational cohort study
Source: Med J Aust. 2025 May 19;222(10):504–9. doi: 10.5694/mja2.52673 (PMC12126968; doi:10.5694/mja2.52673)
Supplement: Supplementary file 1 — Supplementary results [file MJA2-222-504-s001.pdf]

MJA
The Medical Journal of Australia

Supporting Information

Supplementary results

**This appendix was part of the submitted manuscript and has been peer reviewed.
It is posted as supplied by the authors.**

Appendix to: Delardes B, Tofte Gregers MC, Nehme E, et al. Smartphone-activated volunteer responders and survival to discharge after out-of-hospital cardiac arrests in Victoria, 2018–23: an observational cohort study. *Med J Aust* 2025; doi: 10.5694/mja2.52673.

Supplementary results

Table 1. Attendance by smartphone activated volunteer responders and bystander interventions and patient survival: unadjusted logistic regression analyses

	Odds ratio (95% confidence interval)			
**Smartphone-activated volunteer responders**	**Bystander cardiopulmonary resuscitation**	**Bystander defibrillation***	**Any return of spontaneous circulation**	**Survival to discharge**
None	1	1	1	1
Arrived after emergency medical services	1.22 (0.999–1.50)	2.33 (0.98–5.57)	1.08 (0.91–1.29)	1.25 (0.98–1.61)
Arrived before emergency medical services	7.55 (5.00–11.4)	17.0 (10.7–27.0)	1.09 (0.91–1.30)	1.16 (0.90–1.50)

* Patients with initially shockable rhythms only; precipitating event was medical in all such cases.

2. Sensitivity analysis

A total of 1118 patients were included in the sensitivity analysis, 559 (50.0%) of whom received a SAVR response prior to EMS arrival and 559 (50.0%) of whom did not (table 1). In the sensitivity analysis, patients who received a SAVR prior to EMS arrival were more likely to receive bystander CPR (odds ratio [OR], 7.94; 95% confidence interval [CI,] 5.02–12.6) and to survive to discharge (OR, 1.56; 95% CI, 1.06–2.31) than those who did not (table 2). No patients in the matched cohort received bystander defibrillation, but 7.0% of patients with a SAVR arriving prior to EMS received bystander defibrillation (P<0.001).

Table 2. Baseline characteristics of patients attended by smartphone-activated volunteer responders prior to emergency medical services arrival and of propensity score-matched patients

Characteristic	All patients	Smartphone-activated volunteer responders arrived before emergency medical services	Matched cohort	Standard mean difference
Number of cases	1118	559	559	
Age (years), median (IQR)	68 (54–78)	69 (55–77)	68 (54–78)	-0.02
Gender (men)	819 (73.3%)	410 (73.2%)	409 (73.4%)	<0.01
Remoteness (metropolitan)	636 (56.9%)	320 (57.3%)	316 (56.5%)	-0.01
Event location (residence)	901 (80.6%)	452 (80.9%)	449 (80.3%)	-0.01
Witnessed	594 (53.1%)	300 (53.7%)	294 (52.6%)	-0.02
Presumed aetiology (medical)	1087 (97.2%)	543 (97.1%)	544 (97.3%)	0.01
Initial shockable rhythm	9.7 (7.6–12.9)	10.0 (7.8–13.7)	9.5 (7.4; 12.3)	-0.08
Year				<0.01
2018	26 (2.3%)	13 (2.3%)	13 (2.3%)	
2019	156 (14.0%)	78 (14.0%)	78 (14.0%)	
2020	72 (6.4%)	36 (6.4%)	36 (6.4%)	
2021	288 (25.8%)	144 (25.8%)	144 (25.8%)	
2022	364 (32.6%)	182 (32.6%)	182 (32.6%)	
2023	212 (19.0%)	106 (19.0%)	106 (19.0%)	

IQR = interquartile range.

Table 3. Outcomes for patients attended by smartphone-activated volunteer responders prior to emergency medical services arrival and of propensity score-matched patients

Characteristic	Overall cohort	Smartphone-activated volunteer responders arrived before emergency medical services	Matched cohort	Odds ratio (95% CI)
Number of cases	1118	559	559	—
Bystander cardiopulmonary resuscitation	953 (85.2%)	536 (95.9%)	417 (74.6%)	7.94 (5.02–12.6)
Bystander defibrillation	39 (3.5%)	39 (7.0%)	0	—
Any return of spontaneous circulation	374 (33.5%)	206 (36.9%)	168 (30.1%)	1.36 (1.06–1.74)
Survival to discharge	117 (10.6%)	70 (12.7%)	47 (8.5%)	1.56 (1.06–2.31)

CI = confidence interval.

STROBE Statement—Checklist of items that should be included in reports of cross-sectional studies

Note: The page numbers refer to the submitted manuscript, not the published article or its supporting information file.

	Item No	Recommendation	Page No
**Title and abstract**	1	(*a*) Indicate the study's design with a commonly used term in the title or the abstract	Page 1
		(*b*) Provide in the abstract an informative and balanced summary of what was done and what was found	Page 2
**Introduction**			
Background/rationale	2	Explain the scientific background and rationale for the investigation being reported	Page 4
Objectives	3	State specific objectives, including any prespecified hypotheses	Page 4
**Methods**			
Study design	4	Present key elements of study design early in the paper	Page 4
Setting	5	Describe the setting, locations, and relevant dates, including periods of recruitment, exposure, follow-up, and data collection	Page 4 – 6
Participants	6	(*a*) Give the eligibility criteria, and the sources and methods of selection of participants	Page 4
Variables	7	Clearly define all outcomes, exposures, predictors, potential confounders, and effect modifiers. Give diagnostic criteria, if applicable	Page 6 – 7
Data sources/ measurement	8*	For each variable of interest, give sources of data and details of methods of assessment (measurement). Describe comparability of assessment methods if there is more than one group	Page 6
Bias	9	Describe any efforts to address potential sources of bias	NA
Study size	10	Explain how the study size was arrived at	Page 4
Quantitative variables	11	Explain how quantitative variables were handled in the analyses. If applicable, describe which groupings were chosen and why	Page 6 – 7
Statistical methods	12	(*a*) Describe all statistical methods, including those used to control for confounding	Page 7
		(*b*) Describe any methods used to examine subgroups and interactions	Page 7
		(*c*) Explain how missing data were addressed	Page 7
		(*d*) If applicable, describe analytical methods taking account of sampling strategy	NA
		(*e*) Describe any sensitivity analyses	NA
**Results**			
Participants	13*	(a) Report numbers of individuals at each stage of study—eg numbers potentially eligible, examined for eligibility, confirmed eligible, included in the study, completing follow-up, and analysed	Figure 1
		(b) Give reasons for non-participation at each stage	Figure 1
		(c) Consider use of a flow diagram	Figure 1
Descriptive data	14*	(a) Give characteristics of study participants (eg demographic, clinical, social) and information on exposures and potential confounders	Table 1; Page 8
		(b) Indicate number of participants with missing data for each variable of interest	Table 1
Outcome data	15*	Report numbers of outcome events or summary measures	Table 2; Page 8-9
Main results	16	(*a*) Give unadjusted estimates and, if applicable, confounder-adjusted estimates and their precision (eg, 95% confidence interval). Make clear which confounders were adjusted for and why they were included	Table 2; Table 3; Page 8-9

		(*b*) Report category boundaries when continuous variables were categorized	Table 2; Table 3; Page 6
		(*c*) If relevant, consider translating estimates of relative risk into absolute risk for a meaningful time period	NA
Other analyses	17	Report other analyses done—eg analyses of subgroups and interactions, and sensitivity analyses	NA
**Discussion**			
Key results	18	Summarise key results with reference to study objectives	Page 9
Limitations	19	Discuss limitations of the study, taking into account sources of potential bias or imprecision. Discuss both direction and magnitude of any potential bias	Page 11
Interpretation	20	Give a cautious overall interpretation of results considering objectives, limitations, multiplicity of analyses, results from similar studies, and other relevant evidence	Page 9 - 11
Generalisability	21	Discuss the generalisability (external validity) of the study results	Page 11
**Other information**			
Funding	22	Give the source of funding and the role of the funders for the present study and, if applicable, for the original study on which the present article is based	Page 1

*Give information separately for exposed and unexposed groups.

Note: An Explanation and Elaboration article discusses each checklist item and gives methodological background and published examples of transparent reporting. The STROBE checklist is best used in conjunction with this article (freely available on the Web sites of PLoS Medicine at http://www.plosmedicine.org/, Annals of Internal Medicine at http://www.annals.org/, and Epidemiology at http://www.epidem.com/). Information on the STROBE Initiative is available at www.strobe-statement.org.
